# Reference Genes for Quantitative Gene Expression Studies in Multiple Avian Species

**DOI:** 10.1371/journal.pone.0099678

**Published:** 2014-06-13

**Authors:** Philipp Olias, Iris Adam, Anne Meyer, Constance Scharff, Achim D. Gruber

**Affiliations:** 1 Institute of Veterinary Pathology, Freie Universität Berlin, Berlin, Germany; 2 Institute of Biology, Department of Animal Behavior, Freie Universität Berlin, Berlin, Germany; Utrecht University, Netherlands

## Abstract

Quantitative real-time PCR (qPCR) rapidly and reliably quantifies gene expression levels across different experimental conditions. Selection of suitable reference genes is essential for meaningful normalization and thus correct interpretation of data. In recent years, an increasing number of avian species other than the chicken has been investigated molecularly, highlighting the need for an experimentally validated pan-avian primer set for reference genes. Here we report testing a set for 14 candidate reference genes (*18S*, *ABL*, *GAPDH*, *GUSB*, *HMBS*, *HPRT*, *PGK1*, *RPL13*, *RPL19*, *RPS7*, *SDHA*, *TFRC*, *VIM*, *YWHAZ*) on different tissues of the mallard (*Anas platyrhynchos*), domestic chicken (*Gallus gallus domesticus*), common crane (*Grus grus*), white-tailed eagle (*Haliaeetus albicilla*), domestic turkey (*Meleagris gallopavo* f. *domestica*), cockatiel (*Nymphicus hollandicus*), Humboldt penguin (*Sphenicus humboldti*), ostrich (*Struthio camelus*) and zebra finch (*Taeniopygia guttata*), spanning a broad range of the phylogenetic tree of birds. Primer pairs for six to 11 genes were successfully established for each of the nine species. As a proof of principle, we analyzed expression levels of 10 candidate reference genes as well as *FOXP2* and the immediate early genes, *EGR1* and *CFOS*, known to be rapidly induced by singing in the avian basal ganglia. We extracted RNA from microbiopsies of the striatal song nucleus Area X of adult male zebra finches after they had sang or remained silent. Using three different statistical algorithms, we identified five genes (*18S, PGK1, RPS7, TFRC, YWHAZ*) that were stably expressed within each group and also between the singing and silent conditions, establishing them as suitable reference genes. In conclusion, the newly developed pan-avian primer set allows accurate normalization and quantification of gene expression levels in multiple avian species.

## Introduction

The zebra finch (*Taeniopygia guttata*) and the domestic chicken (*Gallus gallus* f. *dom.*) have become widely used model organisms for biologists studying neurobiology and behavior, ecology as well as diseases and their transmission [1]–[5]. With both genomes sequenced and technologies adapted from traditional genetic model systems, the finch and the chicken are becoming increasingly amenable to genetic investigation [6]–[9]. The more than 10,000 avian species are known for their extraordinary differences in behavior and physiology. This offers opportunities to find a suitable species to address particular questions about the genetic background of migration, mating systems, parental care, flight, niche partition, vocal behavior or cognition, to name just a few.

Quantitative real-time polymerase chain reaction (qPCR) is currently the most economic, efficient and reliable method to measure gene expression levels in low to medium throughput approaches. It is sensitive, specific and reproducible even with limited mRNA copy numbers [10]. As in any quantitative study, it is necessary to correct for sample to sample variations in order to obtain reliable results. The most commonly used method is normalization to the expression of an internal control gene, also called reference gene. Hypothetically, the ideal reference gene is expressed at stable levels irrespective of tissue type, species, treatment, metabolism or sampling conditions. To date, no such ideal gene has been found and most likely does not exist, thus it is becoming clear that reference genes need to be established for each new experimental design, controlling for the numerous variables which might bias the results, i.e. number of cells and their transcriptional activity, RNA quality and reverse transcriptase efficiency [11]. In recent avian gene expression experiments, genes such as *18S* (18S ribosomal RNA), *ACTB* (beta actin) and *GAPDH* (glyceraldehyde 3-phosphate dehydrogenase) have been used most frequently [12]–[17] but several studies have shown that using single or inappropriate reference genes for normalization may dramatically bias the results of mRNA copy number quantification [10], [18]–[20].

For mammals, multiple sets of reference genes have been published [21]–[25] but are of limited use for non-mammalian vertebrates including birds due to the phylogenetic distance. Certainly, the advent of Next Generation Sequencing and the now fully sequenced genomes of the domestic chicken, domestic turkey (*Meleagris gallopavo* f. *domestica*), zebra finch, duck (*Anas platyrhynchos*) and the collared flycatcher (*Ficedula albicollis*) has made it easier and faster to conduct gene expression studies in these species [7], [9], . However, currently more than 10,000 avian species are known, more and more of which are also investigated at the molecular level [29]–[32]. The tools available to compare those less genetically amenable bird species are highly limited, one problem being the lack of sequence information to design PCR-primers. To date, avian reference genes have been published only for the domestic chicken [33], [34], great tit (*Parus major*) [32], Japanese quail (*Coturnix c. japonica*) [35] and domestic pigeon (*Columba livia* f. *domestica*) [36].

This prompted us to develop a set of pan-avian PCR primers for the amplification of reference genes that can be tested for their suitability to normalize gene expression levels in avian studies involving qPCR analyses. We designed primer pairs for the amplification of 14 candidate reference genes classically used in mammalian research, including *18S*, *ABL* (Abelson murine leukemia viral oncogene homolog), *GAPDH*, *GUSB* (beta glucuronidase), *HMBS* (hydroxymethyl-bilane synthase), *HPRT* (hypoxanthine-guanine phosphoribosyl-transferase 1), *PGK1* (phosphoglycerate kinase 1), *RPL13* (60S ribosomal protein L13), *RPL19* (60S ribosomal protein L19), *RPS7* (40S ribosomal protein S7), *SDHA* (succinate dehydrogenase complex, subunit A), *TFRC* (transferrin receptor protein 1), *VIM* (vimentin) and *YWHAZ* (tyrosine 3-monooxygenase/tryptophan 5-monooxygenase activation protein, zeta polypeptide). For each of the nine avian species investigated in this study we successfully established between six and 11 primer pairs.

To demonstrate the suitability of these genes for normalization purposes, we conducted an experiment investigating singing-induced gene expression changes in a striatal song control region, Area X of zebra finches. We measured the expression of three genes of interest, among them two immediate early genes known to be upregulated by singing [37]–[39], as well as 10 potential reference genes, on samples of birds that sang undirected song (not for a female conspecific) or remained silent for the same amount of time. Using the statistical algorithms geNorm [11], NormFinder [40] and BestKeeper [41] we determined the most stably expressed genes and employed them to normalize the expression of our genes of interest.

## Material and Methods

Animal experiments were carried out in accordance with the guidelines provided and approved by the governmental institutions (LAGeSo, Berlin, Permit Number: T 0298/01). All zebra finch samples originated from a captive breeding colony approved by local authorities (Permit Number: ZH147). The brain of the ostrich was obtained from a slaughterhouse (Winkler, Neuloewenburg, Germany, registration number: DE-BB65011EG). All other birds had been euthanized for animal welfare reasons for causes unrelated to the present study in strict accordance with the German National Animal Protection law (Tierschutzgesetz in der Fassung der Bekanntmachung vom 18. Mai 2006 (BGBl. I S. 1206, ber. S. 1313), last amendment: Artikel 20 G vom 9. Dezember 2010 (BGBl. I S. 1934, 1940 f.)).

### Sample collection

Three sets of tissues were collected. (1) We collected samples from nine phylogenetically distant avian species: The brains from a mallard (*Anas platyrhynchos*), a domestic chicken (*Gallus gallus domesticus*), a white-tailed eagle (*Haliaeetus albicilla*), a domestic turkey (*Meleagris gallopavo* f. *domestica*), a cockatiel (*Nymphicus hollandicus*) and an ostrich (*Struthio camelus*); from a common crane (*Grus grus*) we took blood and from a Humboldt penguin (*Sphenicus humboldti*) the lung with fungal pneumonia. (2) From adult zebra finches we collected brains and gonads (10 females, 10 males, below called ‘tissue dataset’). (3) We collected microbiopsies of AreaX from another 12 adult zebra finches (below called ‘song data set’). All samples were immediately snap frozen after dissection and stored at −80°C until further use.

#### Song data set

Adult male zebra finches were housed in free flight aviaries in our breeding colony and kept alone in sound attenuated chambers overnight. After lights were switched on in the morning at 8 a.m., song was monitored and recorded. Birds that did not sing during the first 30 min were sacrificed (‘silent group’). Birds that started singing (while being alone, called ‘undirected song’) within the first two hours were sacrificed 30 min after the onset of song (‘song group’). Birds were sacrificed by an isoflurane overdose and brains were immediately dissected, cut into hemispheres, embedded in Tissue-Tek O.C.T. compound (Sakura Finetek) and stored at −80°C. To obtain microbiopsies of Area X, hemispheres were mounted on a cryostat (Cryo-Star HM 560 Cryostat, MICROM) and 20 µm sagittal sections were cut from medial to lateral until Area X became visible. Then a 1 mm diameter coring tool (Harris Unicore) was used to mark Area X while still on the block. Subsequently, a 200 µm section was cut and Area X microbiopsies were taken with the coring tool. The frozen biopsy was immediately transferred to dry ice, while the remaining section was transferred into a 4% paraformaldehyde/0.1 M PBS solution for microscopic inspection of targeting. The procedure was repeated until Area X was no longer visible. Microbiopsies were stored at -80°C until further use.

### Selection of candidate reference genes and primer design

Candidate reference genes were selected from previous reports for mammalian species [21]–[25] (Table 1). All primers except *RPL13* [36] were designed *de novo* on homologous gene segments of the chicken and zebra finch derived from GenBank (http://www.ncbi.nlm.nih.gov) and Ensembl (http://www.ensembl.org/index.html) databases using NetPrimer (http://www.premierbiosoft.com/netprimer/netprimer.html). Primers were designed to reside in the open reading frame (ORF) of genes to grant maximal conservation across all birds. ORFs were identified by Biowire Jellyfish 1.5. Criteria for primer design were a predicted melting temperature of 58°C, primer length of 15–25 nucleotides, a guanine-cytosine content of 40–70% and amplicon lengths smaller than 250 base pairs [10], [42]. All primer pairs except those for 18S were designed to span exon-exon boundaries as identified by MEGA4 [43].

**Table 1:** Gene names and primer pairs tested on all nine avian species.

Gene symbol	GenBank accession number *G. gallus*	Primer sequences 5′-3′ (forward/reverse)	Amplicon length range (bp)	Size on genomic level chicken (bp)
***18S***	AF173612	CGAAAGCATTTGCCAAGAAT	98–99	98
		GGCATCGTTTATGGTCGG		
***ABL***	XM_001233811	GCTGCTCGCTGGAACTCC	218	940
		GTGATGTAATTGCTGGGGACC		
***GAPDH***	NM_204305	GTGGTGCTAAGCGTGTTATCATC	269–270	534
		GGCAGCACCTCTGCCATC		
***GUSB***	NM_001039316	GGCAAACTCCTTCCGCAC	222–224	858
		TCATTGGCTACTGACCACATCA		
***HMBS***	XM_417846	CTGAAGAGAATGGGCTGGGA	113–115	1791
		TCTTGGTCTTTGGCACGAAC		
***HPRT***	NM_204848	GATGAACAAGGTTACGACCTGGA	181	1575
		TATAGCCACCCTTGAGTACACAGAG		
***PGK1***	NM_204985	AAAGTTCAGGATAAGATCCAGCTG	167	450
		GCCATCAGGTCCTTGACAAT		
***RPL13***	NM_204999	CCACAAGGACTGGCAGCG	135	434
		ACGATGGGCCGGATGG		
***RPL19***	NM_001030929	CCAACGAGACCAACGAGATC	152–153	629
		CATGTGCCGGCCCTTCC		
***RPS7***	XM_419936	TAGGTGGTGGCAGGAAAGC	156	1773
		TTGGCTTGGGCAGAATCC		
***SDHA***	XM_419054	TTGGTGGACAGAGTCTTCAGTT	238	1821
		GTGTTCTTTGCTCTAAAACGATG		
***TFRC***	NM_205256	GGAACTTGCCCGTGTGATC	111–113	723
		GTAGCACCCACAGCTCCGT		
***VIM***	NM_001048076	GGAACAATGATGCCCTGC	145	761
		GCAAAATTCTCCTCCATTTCAC		
***YWHAZ***	NM_001031343	GTGGAGCAATCACAACAGGC	222–224	326
		GCGTGCGTCTTTGTATGACTC		

### RNA extraction, cDNA synthesis and qPCR

#### All tissues except for Area X tissue samples (‘song data set’)

RNA was extracted and purified from approximately 100 mg of each sample using Trizol (Invitrogen, Carlsbad, CA) or the NucleoSpin RNA II kit (Macherey-Nagel, Düren, Germany) following the manufacturer's recommendations. Concentration and purity of total RNA were determined at 260/280 nm absorbance ratio to be above 1.8 in all cases (NanoDrop 1000 Spectrophotometer, Thermo Fisher Scientific, Wilmington, DE). RNA integrity numbers (RIN) of all tissue samples were measured using the RNA Nano kit on an Agilent 2100 Bioanalyzer (RNA 6000 Nano Kit, Agilent, Santa Clara, CA) and used only if above 8.0. For cDNA synthesis, 100 ng RNA was reverse transcribed at 25°C for 5 min followed by 30 min at 42°C in 20 µl containing 200 U iScript (Bio-Rad, Hercules, CA), 100 nmol MgCl_2_, 50 ng random hexamers, 0.2 mmol DTT, 40 U RNase Out (Promega, Madison, USA) and 10 nmol of each dNTP. The initial tests for successful product amplification were run on the MX 3000P or the CFX96 qPCR detection system using the MX Pro 3.0 (Agilent) or CFX Manager 2.0 (Bio-Rad) software packages, respectively. All subsequent PCR experiments were performed on the CFX96 system. PCR reactions were carried out in 96-well polypropylene plates (Sarstedt, Nümbrecht, Germany) in a volume of 20 µl. 5 µl cDNA was added to 15 µl reaction mix containing 10 µl Brilliant SYBR Green QPCR Master Mix (Applied Biosystems, Carlsbad, CA) and 4.5 pmol of each primer. Cycling conditions were as follows: 10 min at 95°C followed by 40 cycles of 30 sec at 95°C, 1 min at 58°C, and 30 sec at 72°C and a subsequent melting curve analysis. All reactions were run in triplicate and the mean C_t_-values were used for further analysis. If one of the three C_t_-values deviated from the other two by more than 0.5 it was excluded from further analysis or the experiment was repeated. Nuclease-free water was used for the no-template controls. PCR efficiencies (*E*) were calculated using the equation *E* = 10^−1/slope^ by measuring a ten-fold dilution series over five orders of magnitude for each primer pair and for each avian species as indicated in Table 2. Specificity of the primers was confirmed by sequencing and sequence identity was evaluated with BLASTN [44] against coding sequences of the chicken and zebra finch derived from GenBank (Table 3).

**Table 2:** Summary of primer test and efficiency for each primer pair and species.

Species	18S	ABL	GAPDH	GUSB	HMBS	HPRT	PGK1	RPL13	RPL19	RPS7	SDHA	TRFC	VIM	YWHAZ	No. of established primer pairs
**Zebra finch**	**102.5**	**93.9**	70.8	**100.0**	**96.3**	**93.5**	**94.0**	nd	nd	**100.9**	**103.3**	**103.6**	**95.8**	**96.6**	11
**Cockatiel**	nd	89.9	nd	**91.3**	nd	84.4	**96.8**	nt	nt	**92.8**	**95.8**	**92.3**	**95.5**	84.6	6
**White tailed eagle**	nd	**94.0**	nd	**96.4**	**94.4**	89.2	**94.2**	nt	nt	**93.0**	**97.9**	**100.4**	**98.1**	**93.0**	9
**Humboldt penguin**	nd	**94.3**	nd	**93.0**	84.7	87.5	**93.6**	nt	nt	**95.9**	**94.5**	**97.7**	**98.8**	**94.8**	8
**Common crane**	nd	nw	nd	**90.8**	**91.0**	89.0	**94.2**	nt	nt	**94.8**	86.1	**94.6**	**98.8**	**95.8**	7
**Chicken**	**107.6**	**93.3**	72.7	**90.3**	**94.3**	81.2	88.3	nd	nd	89.8	**96.2**	87.8	**100.8**	**99.7**	7
**Turkey**	nd	**92.4**	nd	**96.0**	**98.3**	88.0	**95.9**	nt	nt	**91.3**	**96.8**	**99.4**	**101.0**	**98.8**	9
**Mallard**	nd	nw	nd	83.4	nw	86.7	**94.4**	nt	nt	**97.9**	**91.1**	**95.7**	**99.5**	**94.7**	6
**Ostrich**	nd	**93.1**	nd	**99.3**	**96.9**	85.8	**96.3**	nd	nd	85.3	**98.5**	85.9	**101.8**	**94.9**	7

Numbers refer to the efficiency determined (bold: efficiency ok); nt, primer not tested; nw, primer tested but not working; nd: efficiency not tested.

**Table 3:** Sequence similarity of all amplified products to the chicken (ICGSC Gallus gallus 4.0) and zebra finch (WUGSC 3.2.4/taeGut1) genomes.

Gene symbol	Sequence identity (%) to *G. gallus*/to *T. guttata*								
	*A. platyrhynchos*	*G. gallus*	*G. grus*	*H. albicilla*	*M. gallopavo*	*N. hollandicus*	*S. humboldti*	*S. camelus*	*T. guttata*
***18S***	100/100	100/100	100/100	98/98	100/100	98/98	97/97	100/100	100/100
***ABL***	nw	100/89	nw	95/92	98/88	97/93	97/93	94/89	89/100
***GAPDH***	95/94	100/92	93/95	94/95	98/93	93/96	93/94	94/92	92/100
***GUSB***	91/89	97/86	88/89	91/89	96/87	90/89	90/90	87/90	87/100
***HMBS***	93/89	100/89	88/93	92/96	97/98	92/90	88/92	88/92	89/100
***HPRT***	95/98	100/94	96/98	95/99	97/95	95/98	95/99	95/99	94/100
***PGK1***	92/95	100/92	93/96	93/97	98/92	93/97	93/95	93/96	92/100
***RPL13*** *	nd	100/85	nd	nd	nd	nd	nd	86/-	90/95
***RPL19***	nd	100/86	nd	nd	nd	nd	nd	89/84	85/97
***RPS7***	98/93	100/93	96/93	97/95	100/93	97/93	97/92	97/92	93/100
***SDHA***	93/89	100/90	94/93	95/93	98/91	92/91	93/91	90/90	90/100
***TFRC***	99/87	100/86	86/95	87/90	99/87	89/86	90/93	94/89	86/100
***VIM***	97/90	100/88	93/90	96/91	98/90	95/92	96/90	93/90	88/100
***YWHAZ***	97/95	100/96	96/95	96/97	99/96	97/97	97/97	97/96	96/99

*[36]; nd, not done; nw, not working.

#### Song data set

After microscopic verification of proper targeting, we extracted the RNA from the Area X microbiopsies of each animal individually, resulting in 12 separate RNA extractions. RNA was extracted using the NucleoSpin RNA XS kit (Macherey-Nagel) and quantified using the Qubit RNA assay (life technologies). For cDNA synthesis 70 ng RNA was reverse transcribed in 20 µL using 200 ng random hexamer primers, 10 nmol of each dNTP, 50 mM DTT, 200 U SuperScript III (Invitrogen) and 40 U RNasinPlus (Promega). The temperature program was chosen as follows: 5 min at 65°C, cool down on ice, 5 min 25°C, 45 min at 50°C, 15 min at 72°C. We included reverse transcriptase-free reactions to control for DNA contaminations. All cDNAs were diluted 10-fold with nuclease free water and 5 µL were used in each PCR reaction.

QPCR reactions were carried out in 96-well polypropylene plates (Sarstedt, Nümbrecht, Germany) in a volume of 20 µl and in triplicates. 5 µl cDNA was added to 15 µl reaction mix containing 10 µl KAPA SYBR FAST Universal QPCR mix (Peqlab) and 10 pmol (18 pmol in the case of FoxP2) of each primer. PCRs were run on a MX3005P system (Agilent) with the following cycling conditions: 10 min at 95°C followed by 40 cycles of 30 sec at 95°C, 30 sec at 65°C (64°C for FoxP2) and a subsequent melting curve analysis. Primer pairs for *FOXP2* (CCTGGCTGTGAAAGCGTTTG/ATTTGCACCCGACACTGAGC [8]), *EGR1* (ACTTCATCATCGCCATCCTC/TGGAATTGGGAAATGTTGGT) and *CFOS* (AGCTGGAGGAGGAGAAGTCC/CTCCTCGGAGAAGCACAACT) were designed for the zebra finch only.

### Analysis of expression stability

The stability of expression was evaluated for 10 zebra finch candidate reference genes (*18S, GUSB, HMBS, HPRT, PGK1, RPS7, SDHA, TFRC, VIM* and *YWHAZ*) on samples from 10 female and 10 male brains and six gonads of each sex, and samples from Area X of male zebra finches that sang before sacrifice (n = 5) or were silent (n = 7). Mean C_t_ values from qPCR runs were exported to Excel (Microsoft Excel 2010) and analyzed by the three commonly used statistical algorithms geNorm (version 3.5), NormFinder (version 0.953) and BestKeeper (version 1.0) [11], [40], [41]. For the geNorm analysis, we employed the comparative C_t_ method, taking into account the efficiency of each assay as outlined in the manual. The resulting values were used as input into the geNorm analysis. For the NormFinder analysis we followed the input procedure outlined in [45]. For the BestKeeper analysis the raw C_t_ values were entered into the Excel mask.

The song data set was analyzed for differences in gene expression using the Mann-Whitney-U-test.

## Results

Normalization to reference genes is a crucial step to obtain meaningful and reliable results, when gene expression levels are quantified using qPCR. In this study we aimed to establish and test a set of primer pairs that could ideally be used on the entire avian class. We selected 14 candidate genes (*18S*, *ABL*, *GAPDH*, *GUSB*, *HMBS*, *HPRT*, *PGK1*, *RPL13*, *RPL19*, *RPS7*, *SDHA*, *TFRC*, *VIM*, *YWHAZ*) and designed non-degenerate primer pairs.

### Establishing and testing primer pairs on the avian class

To test our primers, we collected samples from different branches of the avian tree in an attempt to represent phylogenetic diversity. We chose the zebra finch, cockatiel and white tailed eagle to represent land birds, the Humboldt penguin for water birds, the common crane for the gruiformes, the chicken, turkey and mallard for gallanserae and the ostrich for paleognaths. We tested all primer pairs except *RPL13* and *RPL19* on all species. The performance of each primer pair was evaluated for each species separately and was considered successful if the following criteria were met:

1. Amplification of a single product indicated by a single peak in the melting curve analysis
2. Sequence of the PCR product confirming amplification from the proper gene
3. Efficiency of amplification between 90 and 110%.

**Figure 1:**
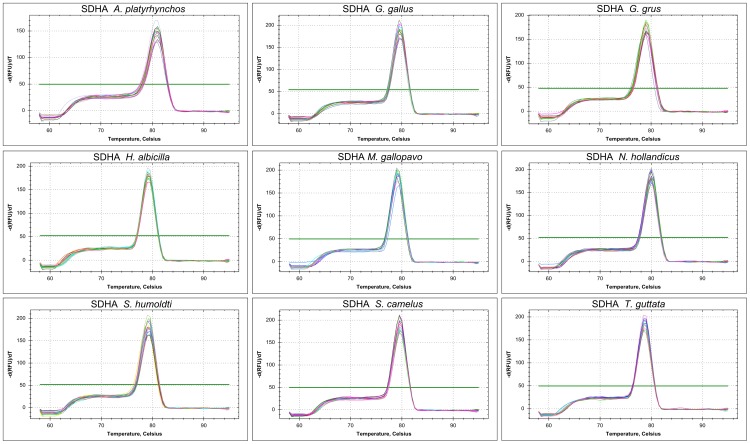
Specificity of primers for *SDHA* in qRT-PCR of nine avian species. Melting curves of five dilutions run in triplicates are shown with one panel per species. Species names are indicated in the title of each panel.

**Figure 2:**
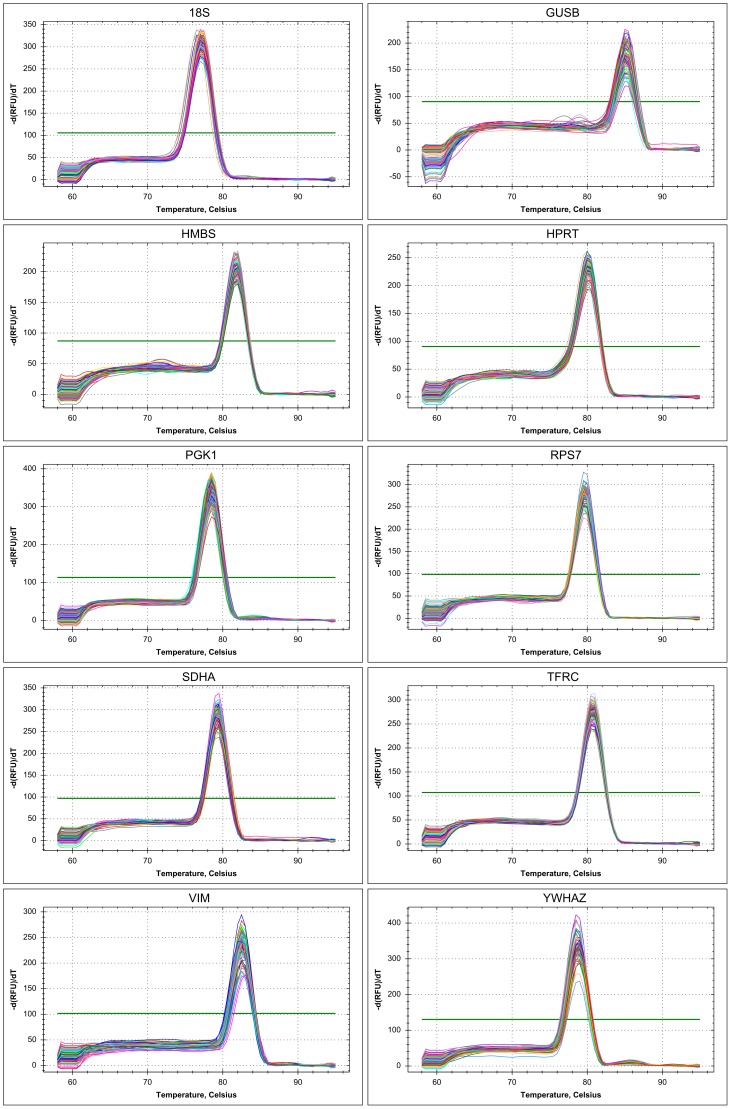
Specificity of 10 reference genes of the zebra finch with single peaks in melting curves. All amplifications were run in triplicate on 32 tissue samples.

Concerning the first criterion, a single product was amplified by most primer pairs in most species (Figure 1, 2; Table 2), except for the following cases. The *ABL* primers did not yield any product on the mallard and crane cDNA but worked on the other species. *RPL13* and *RPL19* were only tested on zebra finch, chicken and ostrich samples because the melting curve analysis suggested amplification of more than one product. However, when we sequenced those samples we obtained clean traces with low background and no sign of more than one template in the sequencing reaction. Likewise, the melting curve of the *HMBS* amplicon on the mallard and cockatiel cDNA also suggested unspecific amplification but sequencing yielded the expected sequence.

With regard to the second criterion, sequencing of the PCR products confirmed the specificity of the amplification in all cases (Table 3; sequences are provided in Document S1). Sequence similarity to the zebra finch and chicken was higher than 84% and 85% in all cases, respectively.

Regarding the third criterion, we determined the amplification efficiency of each primer pair for each species (Table 2). For each avian species the amplification efficiencies of eight to 12 reference genes were determined and ranged between 70.8% and 107.6% (for details see Table 2). We did not determine the efficiency on all species for the *GAPDH* primers as the efficiency on the zebra finch and chicken samples was too low to be considered for the use in gene expression studies. Taken together we established between six and 11 primer pairs for each species.

### Expression stability analysis of candidate reference genes for normalization in the zebra finch

**Figure 3:**
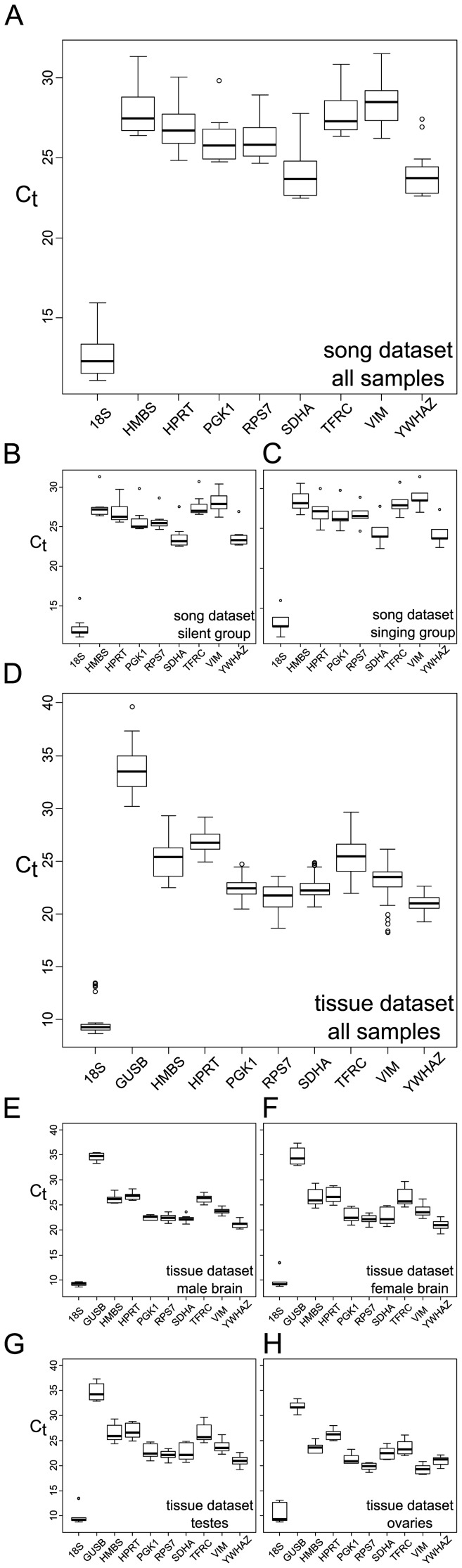
Expression levels of candidate reference genes for the zebra finch song (A–C) and tissue dataset (D–H). Values are given as cycle threshold numbers (C_t_ values) in (A) all Area X samples combined (n = 12), (B) silent group only (n = 7), (C) singing group only (n = 5), (D) tissue dataset all samples (n = 32), (E) male brain only (n = 10), (F) female brain only (n = 10), (G) testes only (n = 6) and (H) ovaries only (n = 6).

To test if the established primers could be used for normalization in a proof of principle experiment, we conducted two independent screens with 10 candidate genes (*18S, GUSB, HMBS, HPRT, PGK1, RPS7, SDHA, TFRC, VIM* and *YWHAZ*) on zebra finch tissues (Figure 3).

1. Microbiopsies of the striatal song nucleus Area X, either taken 30 min after the onset of undirected singing or of a non-singing control (henceforth called “song data set”).
2. Zebra finch entire brains (female and male separately), testes and ovaries.

We determined the expression stability of the 10 genes in both screens using three different algorithms: geNorm [11], NormFinder [40] and BestKeeper [41]. Table 4 and Table 5 display the rankings derived from each of the algorithms for the two data sets. All raw data were prepared for input into the three software applications as described in the methods section. We excluded *GUSB* from the analysis in the song data set, as it was expressed below the detection limit of our assay.

**Table 4:** Stability ranking of expression analysis for the song data set with three different statistical methods.

	Ranking		
Gene	geNorm	Normfinder	BestKeeper
***18s***	**1**	**2**	5
***HMBS***	6	7	5
***HPRT***	8	9	5
***PGK1***	**2**	**3**	5
***RPS7a***	7	5	**1**
***SDHA***	**3**	6	5
***TFRC***	5	**4**	2^*^
***VIM***	9	8	**2**
***YWHAZ***	**4**	**1**	**2**

The first four ranked genes are shown in bold. Asterisks indicate which gene was chosen for normalization in case of multiple genes with one rank.

**Table 5:** Results of testing the stability of expression analysis for the four different zebra finch tissues and three different statistical methods.

Gene	All			Brain			Gonads			Brain male			Brain female			Testes			Ovaries		
	gN	Nf	BK	gN	Nf	BK	gN	Nf	BK	gN	Nf	BK	gN	Nf	BK	gN	Nf	BK	gN	Nf	BK
***18s***	7	8	9	9	9	9	8	8	9	9	9	8	9	9	10	9	8	8	9	8	9
***GUSB***	10	9	7	10	10	10	10	9	8	10	10	10	10	10	9	10	10	10	10	10	10
***HMBS***	6	7	8	5	6	7	6	7	7	6	6	6	5	5	7	**1**	**1**	5	**1**	**2**	**4**
***HPRT***	**2**	**3**	**2**	**1**	**1**	**3**	**3**	**3**	**4**	**1**	**1**	**1**	**2**	**2**	**4**	**3**	5	**2**	**2**	**1**	**1**
***PGK1***	**1**	**1**	**1**	**4**	**4**	**3**	**4**	**4**	6	8	8	**2**	**1**	**1**	**3**	**2**	**4**	**4**	5	**4**	6
***RPS7a***	**3**	**2**	6	7	7	**2**	5	5	**2**	**4**	**3**	5	8	8	**2**	5	6	6	7	7	**3**
***SDHA***	**4**	**4**	**4**	**2**	**2**	5	**1**	**2**	**1**	**3**	5	**4**	**3**	**3**	6	6	**3**	9	**4**	**3**	6
***TFRC***	8	6	5	8	8	7	**2**	**1**	5	7	7	9	7	6	8	7	**2**	7	6	6	8
***VIM***	8	10	9	**3**	**3**	**1**	9	10	10	5	**4**	**3**	**4**	**4**	**1**	8	9	**2**	**3**	5	**2**
***YWHAZ***	5	5	**2**	6	5	6	7	6	**2**	**2**	**2**	6	6	7	4	**4**	7	**1**	8	9	5

Samples were either analyzed together (all), by tissue type (brains, gonads) or separately.

The first four ranked genes are shown in bold. gN, geNorm; Nf, Normfinder: BK, BestKeeper.

#### GeNorm

The geNorm program is a Visual Basic application (VBA) tool for Microsoft Excel [11]. The algorithm assumes that the ratio of two reference genes is unaffected by any experimental treatment or condition. It calculates the pairwise variation of each gene with all other control genes. The stability value M for a gene is the average of all pairwise variations of this particular gene. By stepwise exclusion of the gene with the highest M values, the two most stable reference genes are determined. One critical assumption of the algorithm is that the tested genes are not co-regulated [46]. If they are, they will falsely be considered as suitable reference genes, because of their low co-variation.

In the case of the song dataset, geNorm analysis recommended *18S* and *PGK1* as the most stably expressed genes (Table 4). In contrast, the *18S* gene was one of the least stably expressed genes in all four tissues tested (Figure 3, Table 5).

Besides determining which genes are most suited as reference genes, the geNorm algorithm also gives a recommendation on how many genes should be used for normalization. A Vn/n+1 pairwise variation value below 0.15 indicates that n genes are sufficient for normalization and the gene n+1 should not be included. For the song dataset the V2/3 value (0.079) was already below 0.15 indicating that two genes are sufficient for normalization. For the other tissues the same was true if the tissues were analyzed separately. When all four tissues were analyzed together, three genes were recommended.

#### NormFinder

The NormFinder program, also a VBA based Microsoft Excel tool, is regarded as the most reliable of the three algorithms we employed [40]. In contrast to the other two approaches, NormFinder takes the experimental groups from which samples are drawn into account [46]. The algorithm distinguishes between variation across samples irrespective of the experimental condition, and variation across experimental groups due to experimental condition. To determine the most suited reference genes, NormFinder analyzes the inter- and intra-group variability and calculates a so called “stability value” which takes both measures into account. Consequently, the genes which are most suited as reference genes have the lowest stability values.

For the song dataset *YWHAZ* and *18S* were recommended as reference genes, though *18S* alone did not have the second lowest stability value, but the two genes together had a very low stability value. For the different tissues the results again differed (Table 5). One interesting aspect here was that *YWHAZ* was the second most stable gene in the male brain but only ranked as the seventh most stable gene in the female brain, hinting towards a sexually dimorphic expression in the brain.

#### BestKeeper

The BestKeeper Excel template offers several measures to detect the most stable genes [41]:

1. The standard deviation (SD) of the C_t_s of all samples for one gene.
2. The correlation coefficient with the BestKeeper Index. The BestKeeper Index for each sample is the geometric mean of the C_t_s of all reference genes.
3. The coefficient of variation of a potential reference gene.

The BestKeeper does not indicate how to weigh the importance of these three measures, leaving it up to the experimenter to choose which values to consider most relevant. As a guideline, the authors recommend to exclude all genes with an SD>1.5. However, in the song dataset all candidate genes displayed SDs higher than 1.5, consistent with a previous report finding that the BestKeeper algorithm may be better suited for analysis of gene expression in homogeneous cell populations rather than complex tissues [46]. Given that previous studies used various selection criteria, for example SD only [46], R^2^ only [47], an unspecified combination of SD+CV [48] and in one case a self-designed decision criterion [49], we decided to rank all potential reference genes based on each of the values separately and then calculate the mean of the rankings to determine the final rank of a gene. Employing this method, *RPS7*, *TFRC*, *VIM* and *YWHAZ* were the four most stably expressed genes. We decided to use *RPS7* and *TFRC* for normalization as *TRFC*, unlike *VIM* and *YWHAZ*, was equally stable in all three values contributing to the final ranking (Table 4). For the other tissues the BestKeeper ranking largely agreed with the ranking of the other two programs (Table 5).

### Reference gene validation

To evaluate the usefulness of the reference genes suggested by our data analysis, we measured the expression of *EGR1*, *CFOS* and *FOXP2* genes in the song dataset. It is known that *EGR1* and *CFOS* mRNA is upregulated in a sexually dimorphic subregion of the avian striatum, Area X, after the bird sings undirected song for at least 30 minutes [38], [39]. *FOXP2* is downregulated by undirected singing that lasts for two hours. Whether *FOXP2* is also regulated as early as 30 minutes after the onset of song is currently unknown [50].

**Figure 4:**
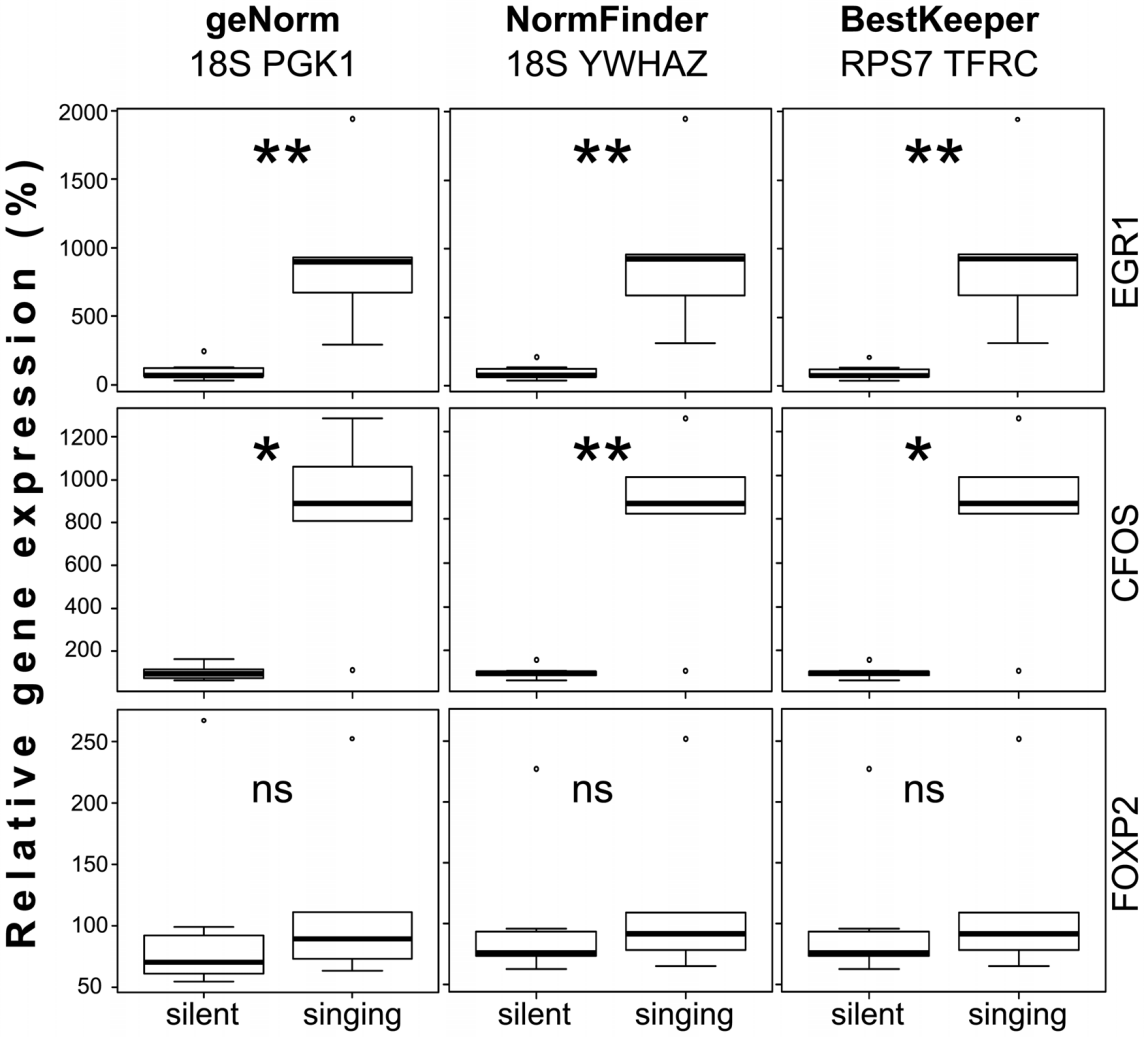
Singing induced gene regulation in Area X of adult male zebra finches. Relative gene expression levels for EGR1, CFOS and FOXP2 were calculated by normalizing to the geometric mean of the two genes recommended by each of the three different algorithms. Relative expression values of the singing group are compared to the silent group. Statistical differences were calculated using the Mann Whitney U test and are indicated in the diagram (p<0.05 *, p<0.01 **, no significant difference ns).

We normalized the expression of each of our genes of interest to the geometric mean of the two best genes recommended by each algorithm. Regardless of which genes we chose for normalization, we detected a significant upregulation of *CFOS* and *EGR1* by more than 7-fold in the singing animals compared to the silent controls (Figure 4). We failed to detect such a significant result for *FOXP2*. The upregulation of *CFOS* was most significant if normalized to *18S* and *YWHAZ* as suggested by NormFinder. The results for *EGR1* and *FOXP2* did not differ much between the different normalizations. These results support the suitability of our pan-avian primer set to identify appropriate reference genes for gene expression studies on avian cells and tissues.

## Discussion

Gene expression studies in diverse bird species are used to explore the molecular mechanisms underlying physiology and behavior. Due to the richness of species, the comparative approach to understand the evolution of particular traits is especially promising in the avian lineage. As more genomes are being sequenced, this endeavor becomes increasingly feasible. Frequently quantitative gene expression data derived from qPCR experiments are used as a first step to probe functional consequences of gene activity [8], [51], [52]. For these data to be meaningful it is essential to choose an appropriate method to normalize the expression level of the gene of interest.

Because the genomes of only very few avian species have been fully explored to date [7], [9], [26], [27], it can be difficult and time consuming to establish primers for qPCR which work on all species under study. Systematic evaluations of reference genes for avian species other than the chicken [33], [53], [54] are scarce. The present study aimed to establish primers for a set of 14 candidate reference genes and to test whether they can be used to normalize gene expression in qPCR experiments on any given avian species. First we tested the adequacy of the primer pairs for each of our candidate genes on nine different avian species spanning the phylogenetic tree from the ostrich to the zebra finch. Subsequently, we evaluated the suitability of the tested genes as reference genes on samples from zebra finch brain microbiopsies. Moreover, we examined the expression levels of the candidate reference genes in gonads and brains from male and female zebra finches.

Of the 14 primer pairs tested, six to 11 turned out to match all our quality criteria on each species. Even though the *GAPDH* primers amplified properly on all species tested, we excluded the primers because of the low amplification efficiency for the zebra finch and chicken. Although we have not yet tested the primers *RPL13* and *RPL19* on all nine avian species of this study, our initial results from the chicken, ostrich and zebra finch indicate that both could serve as additional sets to the established ones. Additionally, we previously used *RPL13* and *RPL19* for normalization in the domestic pigeon (*Columba livia* f. *dom*.) [36] and the cockatiel (A. Meyer, personal communication).

The overall evaluation of all our analyses revealed that *PGK1* was consistently among the most stably expressed genes. Notably, the results calculated by the three algorithms employed (BestKeeper, geNorm, NormFinder) varied considerably between different tissues and sexes. No single gene ranked as the most stable one in all tissues tested. This emphasizes the importance of determining the most reliable reference genes for each new experimental design [11]. This point gains even more emphasis when comparing the results of the stability analysis in the song dataset on the one hand and the total brain samples from male zebra finches on the other hand. *HPRT1* was the most stable gene when analyzing the entire male brain but the least stably expressed gene in Area X under the conditions tested (even though Area X is part of the brain and thus contributes to the first result). Another example of how differently genes can be expressed in similar tissues is *VIM* in the gonad samples and *PGK1* in the male and female brain samples. While *VIM* is stably expressed in ovaries, it is one of the least stably expressed genes in testes. Likewise, *PGK1* is one of the most stably expressed genes in the female brain, while it is among the least stable genes in the male brain. The sexually dimorphic variation of *PGK1 and YWHAZ* in the zebra finch brain might point towards hormonal regulation of gene expression or even a functional role in, e.g. the song system, which is fully developed only in male zebra finches.

As expected, 18S was the most abundant mRNA and *GUSB* the least abundant mRNA in all samples tested (Figure 3). In fact, in the song data set *GUSB* expression was below the detection limit of the qPCR-assay. The use of 18S to normalize gene expression in a qPCR experiment has been heavily debated in the past [20], [55], [56]. A control gene should be expressed at roughly the same level as the gene of interest to minimize the influence of the technical error [57]. It thus might be advisable to avoid using *18S* as reference gene for all but the most abundantly expressed genes. Additionally, *18S* might not be a suitable reference gene as it is produced by RNA Polymerase I whereas synthesis of mRNA is performed by RNA Polymerase II [20]. *18S* is not polyadenylated and one thus needs to be cautious to only use it if the experimental conditions allow it. For example, if the RNA for the experiment was extracted via oligo(dT) purification or reverse transcribed using oligo(dT)s, *18S* should clearly not be used as reference gene. In spite of this criticism, *18S* has repeatedly been used to normalize gene expression in qPCR experiments and we thus decided not to exclude it from our results [56], [58]–[60].

In conclusion, the practical and easy-to-use pan-avian reference gene primer panel will greatly facilitate molecular research in multiple avian species.

## Supporting Information

Document S1 FASTA file of all sequencing results yielded from the PCR products during establishing the primer pairs on nine different species.
(FASTA)
